# RiceAtlas, a spatial database of global rice calendars and production

**DOI:** 10.1038/sdata.2017.74

**Published:** 2017-05-30

**Authors:** Alice G. Laborte, Mary Anne Gutierrez, Jane Girly Balanza, Kazuki Saito, Sander J. Zwart, Mirco Boschetti, M.V.R. Murty, Lorena Villano, Jorrel Khalil Aunario, Russell Reinke, Jawoo Koo, Robert J. Hijmans, Andrew Nelson

**Affiliations:** 1 Social Sciences Division, International Rice Research Institute (IRRI), Los Baños 4031, Laguna, Philippines; 2 Sustainable Productivity Enhancement Program, Africa Rice Center (AfricaRice), 01 BP 2031, Cotonou, Benin; 3 Institute for Electromagnetic Sensing of the Environment, Italian National Research Council, Via Bassini 15, Milan 20133, Italy; 4 Plant Breeding Division, International Rice Research Institute (IRRI), Los Baños 4031, Laguna, Philippines; 5 Environment and Production Technology, International Food Policy Research Institute (IFPRI), Washington, District Of Columbia 20005, USA; 6 Environmental Science and Policy, University of California, Davis, California 95616, USA; 7 Department of Natural Resources, ITC - Faculty of Geo-Information Science and Earth Observation of the University of Twente, PO Box 217, 7500 AE Enschede, The Netherlands

**Keywords:** Agriculture, Geography

## Abstract

Knowing where, when, and how much rice is planted and harvested is crucial information for understanding the effects of policy, trade, and global and technological change on food security. We developed RiceAtlas, a spatial database on the seasonal distribution of the world’s rice production. It consists of data on rice planting and harvesting dates by growing season and estimates of monthly production for all rice-producing countries. Sources used for planting and harvesting dates include global and regional databases, national publications, online reports, and expert knowledge. Monthly production data were estimated based on annual or seasonal production statistics, and planting and harvesting dates. RiceAtlas has 2,725 spatial units. Compared with available global crop calendars, RiceAtlas is nearly ten times more spatially detailed and has nearly seven times more spatial units, with at least two seasons of calendar data, making RiceAtlas the most comprehensive and detailed spatial database on rice calendar and production.

## Background & Summary

Rice is the world’s most important food crop. It is harvested from over 163 million ha in more than 100 countries (http://www.fao.org/faostat/en/#home). It is grown in diverse cropping systems and environments—from single crop systems in temperate and tropical regions in both rainfed and irrigated conditions, to intensive monoculture in irrigated areas in the tropics where rice is grown two or three times per year.

Although information on the distribution of global rice production by region and country can be derived from readily available statistics (e.g., http://www.fao.org/faostat/en/#home, http://apps.fas.usda.gov/psdonline/), information on its distribution within a year is often lacking. Rice area and production statistics are available only at the national level for some countries; if these are available at the subnational level, the statistics are often by year and not by season. Linking information on production and area to the crop calendar can help analyze spatio-temporal variation in rice production. This can contribute to an improved ability to answer questions about food security. For example, this information, together with data on climate shocks and rice stocks, can be used to better assess seasonal and geographic variation in rice supply to mitigate shortfalls in rice availability at certain times of the year. Furthermore, information on where and when rice is planted is needed to quantify the potential risk of abiotic and biotic stresses during the rice-growing seasons, and to model the effects of global climate change and technological change on rice yield and production. In summary, a globally complete and spatially explicit rice crop calendar linked to area and production estimates is a valuable global public good.

Several rice crop calendars exist (http://www.fao.org/agriculture/seed/cropcalendar/welcome.do, 1,2,3). Some are limited to a few countries or have regional coverage, whereas others are global. Regional resources include crop calendars for Latin America and the Caribbean^1^ and for Africa (http://www.fao.org/agriculture/seed/cropcalendar/welcome.do), which have information on the planting and harvesting periods of rice and other major crops by agro-ecological zone. The database on rice for Africa includes 26 countries, and that for Latin America includes 24. The calendar for Latin America is outdated, whereas that for Africa does not include many rice-producing countries. Global calendars have been developed recently^2,3^. The calendars by Sacks *et al.*^2^ lack detail at the subnational level, especially in developing countries. The MIRCA2000 (ref. 3) calendar is monthly, gridded, and available for irrigated and rainfed rice, but does not adequately cover rice areas that are cultivated more than once a year. In both global calendars, some areas with rice grown in two seasons have data for only one season. Also, they include only a maximum of two seasons—the main and second season—inadequately covering some of the world’s most important rice areas with three distinct cropping seasons, such as in Bangladesh, where *aman* rice (main rainy season) is harvested in November-December; *boro* (dry season) in April-May; and *aus* in July-August. Parts of Vietnam also have three cropping seasons—winter-spring, spring-summer, and summer-autumn—and this is also the case in parts of China (early and late seasons for double-cropped rice areas; and middle for single-cropped rice areas) and India.

Because of the need to develop a spatially explicit global database of rice calendars that includes detailed information on rice areas with more than one rice crop in a year, we compiled the most detailed available datasets of rice planting and harvesting dates by growing season in all rice-producing countries, and linked the database to subnational production data. ‘RiceAtlas’ provides a spatial and seasonal distribution of the world’s rice production. RiceAtlas contributes to the GEOGLAM (Group on Earth Observations Global Agricultural Monitoring)^4^ initiative and regional partnerships, such as the Asian Rice Crop Estimation and Monitoring initiative (Asia-RiCE), by providing information for agricultural monitoring requirements, satellite data acquisition plans, and global crop outlook.

## Methods

### Rice calendar

The rice calendar in RiceAtlas is based on various published sources such as global and regional datasets, international and national publications, online sources, and unpublished data sources such as expert knowledge. Collaborators from various countries contributed new datasets, which were used to revise or validate the initial database that was compiled from existing sources (Table 1, Supplementary Table 1).

We collected data on the start, peak, and end dates of sowing or transplanting, and the start, peak, and end dates of harvesting of rice for all seasons in all rice-growing countries. In cases where peak planting and harvesting dates were not available, we estimated those to be at the midpoint between the start and end dates. If only the peak planting dates were available, we assumed the start and end dates of planting to be 15 days before and 15 days after the peak date, respectively. The same procedure was used to estimate the start and end dates of harvesting if only peak harvesting dates were available. Planting in a region is not done on a single date but the length of the planting window varies between regions. In the absence of information, we set the planting window to 30 days. This can be revised when better information becomes available. Where available, additional data such as crop establishment method and seedling age for transplanted rice were recorded.

To describe RiceAtlas and compare its calendar with existing regional (http://www.fao.org/agriculture/seed/cropcalendar/welcome.do; ref 1) and global (Sacks *et al.*^2^ and MIRCA2000^3^) datasets, we used the following metrics:

1. Coverage. This refers to the number of rice growing countries with data.
2. Spatial detail. This refers to the number of spatial units for which data is available. To avoid double counting, we merged adjacent spatial units that have the same calendar. In the case of MIRCA2000, rice calendars are available for irrigated and rainfed rice. If both were available for one spatial unit, only the irrigated calendar was considered in the count of the spatial units.
3. Seasonal detail. This is the number of spatial units with calendars for two or more seasons. This is a metric for the temporal completeness of the crop calendar.
4. Resolution. We used measures similar to those used by Deichmann^5^:
5. Overall spatial resolution $=\sqrt{\frac{Land\;area\;(1000\;km^{2})}{Number\;of\;spatial\;units}}$
6. Rice area resolution $=\frac{\mathrm{Rice}\mathrm{area}(1000\mathrm{ha})}{\mathrm{Number}\mathrm{of}\mathrm{spatial}\mathrm{units}}$

### Rice production

We compiled rice production and area (henceforth referred to as production data or production statistics) from various sources such as national statistics agencies, agriculture departments, and the FAO (http://www.fao.org/faostat/en/#home, http://www.fao.org/economic/ess/countrystat/en/; Table 1, Supplementary Table 1). Because available rice production data from different countries do not refer to the same years, we used the average of the last three years of available data, and adjusted these, such that the national production totals match the 2010–2012 average production from FAO (http://www.fao.org/faostat/en/#home). A three-year average retains the recent production level and accounts for interannual fluctuations in production.

### Spatial and seasonal analysis of rice production

Rice production data were linked with the crop calendar data through their locations using administrative boundaries in the GADM database of the Global Administrative Areas (version 2.8; http://www.gadm.org). There were two cases where the compiled data needed adjustment:

1. Different levels of spatial resolution. For example, production statistics were available at one level (e.g., first level subdivisions) and the crop calendar was available at the a more detailed level (e.g., second level subdivisions) (35 countries);
2. Mismatch in seasonal information, for example, the crop calendar reported double cropping, but the production data referred to only a single cropping season (33 countries).

**Figure 1:**
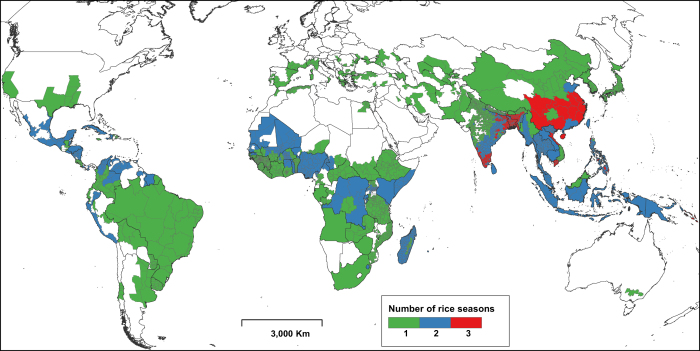
Spatial units covered in RiceAtlas and the number of rice-growing seasons.

**Figure 2:**
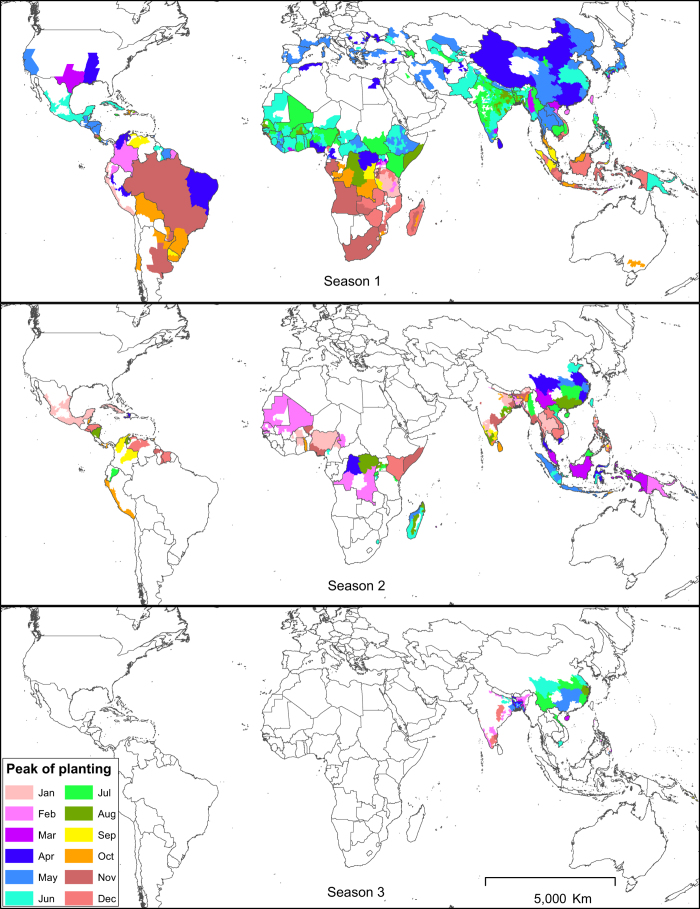
Peak rice planting months by season. Season 1 refers to the main rice-growing season, that is, the season with the highest rice production. Season 2 has the second highest rice production and Season 3 the least.

In cases where the calendar data were spatially more detailed than the production data, we disaggregated the latter using expert knowledge where available, or by assuming equal production over the entire area or season. Conversely, if production data were more detailed than the calendar data, we disaggregated the latter and assumed the same calendar for all disaggregated spatial units. If the production statistics explicitly referred to only a single cropping season but the rice calendar had more than one season, rice production was attributed to the main season only. Because of the differences in the level of detail of available data for both rice calendar and production, the spatial detail of RiceAtlas varies across continents and countries (Figures 1 and 2, Table 2).

**Figure 3:**
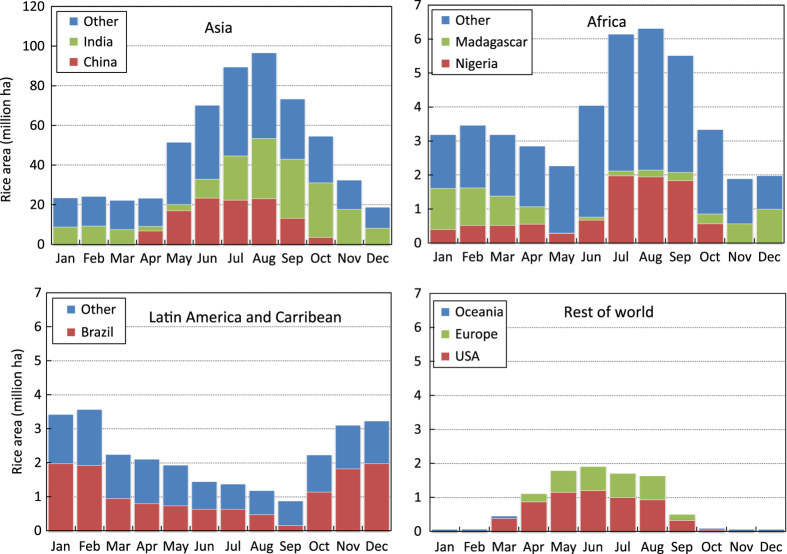
Monthly rice crop area in different geographic regions, 2010-2012.

**Figure 4:**
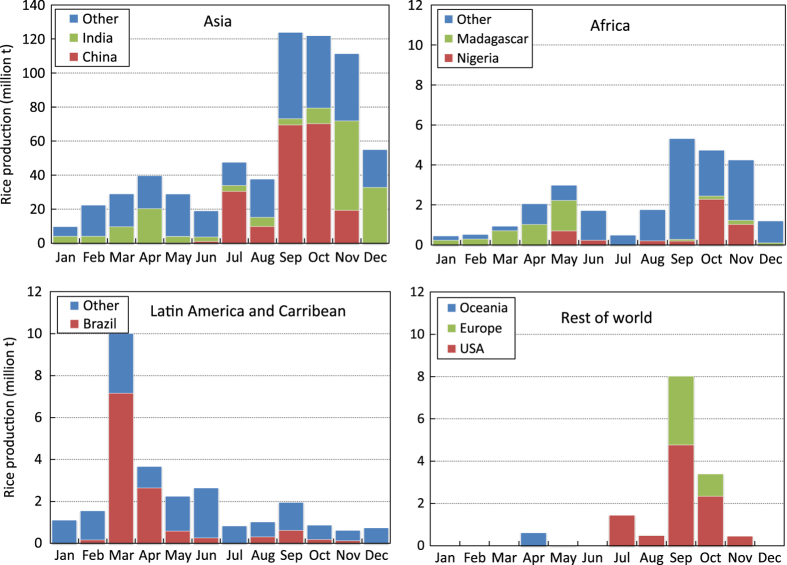
Rice production by geographic region and month of harvest, 2010-2012.

The production data were distributed proportionally to the corresponding months based on sowing or transplanting, and harvesting dates (start, peak, and end) per season, with the peak planting or harvesting months having greater weight and progressively lesser weights for months away from the peak. For example, if the planting window was three months and the peak planting was the middle month, the first and last months were given equal weights of 0.25 each whereas the peak planting month was given 0.5. If production data for a region was available annually but there were two or more known crop seasons in a year in that area, production data were first disaggregated by season (equally, if seasonal data were not available) then distributed to months based on months of harvest. Area data, on the other hand, were distributed based on the months of both planting and harvesting to estimate the monthly area with a standing rice crop. Global monthly rice-growing areas, as well as regional and within-year distribution of rice production at harvest time, show the production peak and lean periods by region (Figures 3 and 4).

### Code availability

R code used to compute for the total planted area or production by month for each country is available from Data Citation 1.

## Data Records

RiceAtlas is a spatial database of rice calendars and production in 115 countries, with its attributes given in Table 3. RiceAtlas version 1.0 can be downloaded from the IRRI Dataverse Repository (Data Citation 1).

RiceAtlas will be improved by including more detailed data for selected countries when these become available and accessible. New versions will be periodically uploaded to the Repository.

## Technical Validation

Expert knowledge was used to validate and correct the crop calendars. More than 50 persons contributed data and/or validated the crop calendars. Digital and analogue country-level maps and tabulations were provided to rice experts to verify the data for countries they were familiar with. Maps and tables were provided to members of the Temperate Rice Research Consortium during their review and planning meeting at IRRI headquarters on 8−9 November 2013. The data from Africa were reviewed by 27 rice scientists during the AfricaRice Science Week on 9−13 February 2015 in Cotonou, Benin. Their comments were addressed and their revisions were included in the current version. All authors validated the crop calendars for their respective regions of expertise.

RiceAtlas v1.0 has 2,725 spatial units in total. It has 2,209 unique contiguous spatial units for the rice calendar and is nearly 10 times more spatially detailed than previously published global rice calendars (Table 4). It has 904 spatial units with data for at least two rice growing seasons. This is almost seven times more than those of published global rice calendars and nearly 10 times more for Asia. RiceAtlas, therefore, greatly improves coverage of intensively cultivated rice areas in the region. In addition, based on comparison among rice calendars in terms of overall spatial resolution and rice area resolution, RiceAtlas is the most comprehensive and detailed global rice calendar currently available.

RiceAtlas will be updated as more detailed data become available and accessible. To date, the effort has concentrated on collecting and validating data for Asia and Africa. Arrangements have been made to update data for Latin America through the International Center for Tropical Agriculture (CIAT) and its network of partners in the region.

The spatial detail of RiceAtlas varies across continents and countries, and for some applications a higher spatial resolution could be desirable. To create a more homogenous and higher spatial resolution database, the available data could be used to build predictive models to downscale the rice calendar data to a higher spatial resolution (e.g., 1 km^2^ grid cells). Such models could use climate data and satellite images. For example, Moderate Resolution Imaging Spectroradiometer (MODIS) images have been used to detect key phenological stages of the rice crop including start of season^6^. The use of time series of satellite images can also allow for the detection of annual variation, changes in cropping intensity and shifts in planting dates.

## Additional Information

**How to cite this article**: Laborte, A. G. *et al.* RiceAtlas, a spatial database of global rice calendars and production. *Sci. Data* 4:170074 doi: 10.1038/sdata.2017.74 (2017).

**Publisher**’**s note**: Springer Nature remains neutral with regard to jurisdictional claims in published maps and institutional affiliations.

## Supplementary Material



Supplementary Table 1

## Figures and Tables

**Table 1:** Main data sources for rice-growing regions*

**Region**	**Calendar**	**Production**
Asia	Various national sourcesIndia: Gumma *et al.*^7^; Rice Knowledge Management Portal (2011); National Food Security Mission, Government of IndiaChina: Van Wart *et al.*^8^	CountrySTAT† FAOSTAT‡, Various national sourcesIndia: DACNET; Department of Agriculture and Extension of the respective states or from Ministry of Agriculture, IndiaChina: China Agricultural Yearbook (various issues, 2009-2011)
Africa	Experts from AfricaRice and its partners, FAO§	CountrySTAT†, FAOSTAT‡Nigeria: CountrySTAT†Madagascar: FAOSTAT‡
Latin America and Caribbean	FAO^1^Brazil: Maclean *et al.*^9^; IBGE, Brazil Rice	FAOSTAT‡
Europe	Various sources	FAOSTAT‡
Rest of the world	Various sources	FAOSTAT‡

*Supplementary Table 1 lists data sources by country.

^†^http://www.fao.org/economic/ess/countrystat/en/.

^‡^http://www.fao.org/faostat/en/#home.

^§^http://www.fao.org/agriculture/seed/cropcalendar/welcome.do.

**Table 2:** Level of detail of RiceAtlas by geographic region.

**Region**	**No. of countries**	**No. of spatial units** *	**Overall spatial resolution (000 km)**	**Rice area per spatial unit (000 ha)**		
	**All**				**With subnational data**	**With two or more seasons**
*Rice calendar*						
Asia	31	17	15	1,121	5	129
Africa	42	25	20	966	5	11
Latin America and the Caribbean	26	11	15	87	15	66
Europe	11	4	0	24	28	30
Rest of the world	5	1	2	11	40	116
Total	115	58	52	2,209	7	74
*Rice production*						
Asia	31	17	15	1,183	5	122
Africa	42	35	20	609	7	17
Latin America and the Caribbean	26	21	15	316	8	18
Europe	11	7	0	36	23	20
Rest of the world	5	1	1	28	25	45
Total	115	77	51	2,172	7	75

*Adjacent spatial units with the same calendar were merged and the values here refer to the number of remaining units.

**Table 3:** Attributes of RiceAtlas.

**Attribute name**	**Unit**	**Description**
*Location information*		
CONTINENT		Continent
ISO		ISO three-letter country code (alpha-3)
COUNTRY		Country name
REGION		Subnational unit: This can be state, province, or district, depending on country
SUBREGION		Subnational unit lower than region
HASC		Hierarchical administrative subdivision codes (HASC) represent names of country subdivisions, such as states, province, and regions
*Crop calendar*		
NAME*		Name of the season
NUM_CROP		Number of distinct rice crop seasons in a year
PLANT_ST*	Day-of-year	Onset of planting period
PLANT_PK*	Day-of-year	Peak planting date
PLANT_END*	Day-of-year	End of planting period
HARV_ST*	Day-of-year	Onset of harvest period
HARV_PK*	Day-of-year	Peak harvest date
HARV_END*	Day-of-year	End of harvest period
MATURITY*	Number of days	Growing period of rice from sowing to harvesting
MATURITYS*	Number of days	Onset of maturity duration range
MATURITYE*	Number of days	End of maturity duration range
SEED_AGE*	Number of days	Age of rice seedlings at the time of transplanting; 0 for direct seeded; −1 for no data
METHOD		Dominant planting method (direct-seeded, transplanted) for all seasons
*Production*		
A_TOTAL	ha	Total rice area
A_S*	ha	Area by season
A_Jan, A_Feb, A_Mar, A_Apr, A_May, A_Jun, A_Jul, A_Aug, A_Sep, A_Oct, A_Nov, A_Dec	ha	Rice area by month
P_TOTAL	t	Total rice production
P_S*	t	Rice production by season
P_Jan, P_Feb, P_Mar, P_Apr, P_May, P_Jun, P_Jul, P_Aug, P_Sep, P_Oct, P_Nov, P_Dec	t	Rice production by month

*The actual attribute names have suffixes: 1 refers to the main season, 2 for second season, and 3 for third season, e.g., PLANT_ST1, PLANT_ST2, PLANT_ST3.

**Table 4:** Level of detail of RiceAtlas and selected global or regional rice calendars.

**Metric/geographic region**	**RiceAtlas**	FAO*	**Sacks** ***et al***.^2^	**MIRCA2000^3^**
Countries covered (number)				
Asia	31	_	22	31
Africa	42	26	30	42
Latin America and the Caribbean	26	24	23	26
Europe	11	_	3	11
Rest of the world	5	_	2	5
World	115	_	80	115
Spatial units (number)†				
Asia	1,121	_	89	94
Africa	966	113	33	42
Latin America and the Caribbean	87	45	24	65
Europe	24	_	3	11
Rest of the world	11	_	8	16
World	2,209	_	157	228
Number of spatial units with at least two seasons				
Asia	630	_	27	64
Africa	239	117	3	27
Latin America	33	39	5	43
Europe	0	_	0	0
Rest of the world	2	_	0	3
World	904	_	35	137
Average resolution (000 km)				
Asia	5	_	16	17
Africa	5	13	24	25
Latin America	15	21	29	18
Europe	28	_	17	42
Rest of the world	40	_	46	33
World	7	_	23	22
Average rice area per spatial unit (000 ha)				
Asia	129	_	1,609	1,537
Africa	11	93	307	252
Latin America and the Caribbean	66	127	238	88
Europe	30	_	124	65
Rest of the world	116	_	221	80
World	74	_	1,027	714

*http://www.fao.org/agriculture/seed/cropcalendar/welcome.do and ref 1.

^†^Calendars were dissolved and the values here refer to the number of contiguous spatial units with unique crop calendars.
