# Supplementary material for: RiceAtlas, a spatial database of global rice calendars and production
Source: Sci Data. 2017 May 30;4:170074. doi: 10.1038/sdata.2017.74 (PMC5448352; doi:10.1038/sdata.2017.74)
Supplement: Supplementary Table 1 [file sdata201774-s2.docx]

### Supplementary information

Supplementary Table 1. Data sources.

| Continent/ Country | Calendar | Production statistics |
| --- | --- | --- |
| ASIA |  |  |
| Afghanistan | FAO: Food and Agriculture Organization of the United Nations, for a world without hunger. FAO Rice Information, Volume 3, December 2002. Retrieved October 31, 2012, from http://www.fao.org/docrep/005/Y4347E/y4347e00.htm#Contents Hijmans, Robert. Crop calendar data (unpublished). | FAOSTAT^1^ |
| Azerbaijan | FAO^3^ | FAOSTAT^1^ |
| Bangladesh | Gumma, M.K., Nelson, A., Thenkabail, P., Singh, A. Mapping rice areas of South Asia using MODIS multitemporal data. *J. Appl. Remote Sens*. **5(1),** 053547. doi:10.1117/1.3619838 (2011). Gumma, Murali Krishna. Ground-truth survey for rice classification in South Asia: Bangladesh. August 2010. Raw data. IRRI, Los Baños. Gateway to Land and Water Information. 2004. Chart 1.3.3: Rice and wheat crop calendar. Retrieved August 10, 2011 from http://www.apipnm.org/swlwpnr/reports/y_sa/z_bd/bdch133.htm MVR Murty. Expert Opinion. Reviewed by Parvesh Kumar Chandna. | FAORAP, RDES (Food and Agricultural Statistics in Asia and the Pacific. Regional Data Exchange Systems);  Bangladesh Bureau of Statistics(BBS) FAOSTAT^1^ |
| Bhutan | Gumma, M.K., Nelson, A., Thenkabail, P., Singh, A. Mapping rice areas of South Asia using MODIS multitemporal data. J. Appl. Remote Sens. 5(1), 053547. doi:10.1117/1.3619838 (2011). MVR Murty. Expert Opinion. | Food and Agriculture Data Network. 2013. CountrySTAT^2^  FAOSTAT^1^ |
| Brunei | Robert Hijmans. Crop calendar data (unpublished). | FAOSTAT^1^ |
| Cambodia | Rice Almanac^6^ Reviewed by Dule Zhao. | Annual Report for Agriculture, Forestry and Fisheries, Ministry of Agriculture, Forestry and Fisheries, Kingdom of Cambodia, various years FAOSTAT^1^ |
| China | Derived from ORYZA^7^ van Wart, J., Kersebaum, K.C., Peng, S., Milner, M., Cassman, K.G. Estimating crop yield potential at regional to national scales. Field Crop. Res. 143, 34–43. doi:10.1016/j.fcr.2012.11.018 (2013). | China Statistical Yearbook National Bureau of Statistics of China  FAOSTAT^1^ |
| East Timor | Derived from ORYZA^7^ Global Information and Early Warning System (GIEWS) & FAO: Food and Agriculture Organization of the United Nations, for a world without hunger. 2012. GIEWS Country Briefs - homepage. Retrieved October 31, 2012, from <http://www.fao.org/giews/countrybrief/index.jsp> | Ministry of Agriculture, Agricultural Statistical Database (BDSP) http://aplikasi.pertanian.go.id/bdsp/index-e.asp FAOSTAT^1^ |
| India | Gumma, M.K., Nelson, A., Thenkabail, P., Singh, A. Mapping rice areas of South Asia using MODIS multitemporal data. J. Appl. Remote Sens. 5(1), 053547. doi:10.1117/1.3619838 (2011). Rice Knowledge Management Portal. 2011. Research Domain: Rice State Wise. Retrieved November 14, 2012 from http://www.rkmp.co.in/research-domain/rice-state-wise National Food Security Mission, Government of India. (n.d.). Crop Calender. Retrieved October 7, 2011, from http://nfsm.gov.in/nfmis/RPT/CalenderReport.aspx MVR Murty. Expert Opinion. Reviewed by Parvesh Kumar Chandna. | Directorate of Rice Development (DACNET). (n.d.). Variety wise, Parentage, year of Notification, Duration, Eco-system, Salient features & recommended for cultivation in different States from 1996 to 2007. Retrieved August 10, 2011 from http://drdpat.bih.nic.in/Downloads/Rice-Varieties-Upto-2007.pdf Parvesh Kumar Chandna Rice Knowledge Management Portal (RKMP) http://www.rkmp.co.in FAOSTAT^1^ |
| Indonesia | Integrated Cropping calendar v 1.8. 2014. http://katam.litbang.pertanian.go.id/main.aspx  Rice Almanac^6^ Derived from ORYZA^7^ Reviewed by Inez. | Ministry of Agriculture, Agricultural Statistical Database (BDSP). 2009. http://aplikasi.pertanian.go.id/bdsp/index-e.asp  FAOSTAT^1^ |
| Iran | Boschetti, Mirco. Eurasia Rice Mapping Slide (unpublished).  Rice Almanac^6^  Hijmans, Robert. Crop calendar data (unpublished). | FAOSTAT^1^ |
| Iraq | World Rice Statistics^5^  Boschetti, Mirco. Eurasia Rice Mapping Slide (unpublished). | FAOSTAT^1^ |
| Japan | Ministry of Agriculture, Forestry and Fisheries. 2010. Status of annual production of rice by prefecture (translated from Japanese to English). Retrieved November 26, 2012 from http://www.maff.go.jp/ | Statistics and Information Department, Minister's Secretariat, Ministry of Agriculture, Forestry and Fisheries. 2009.  FAOSTAT^1^ |
| Kazakhstan | USDA. (n.d.) Kazakhstan Agricultural Overview. Retrieved January 17, 2012, from http://www.pecad.fas.usda.gov/highlights/2010/01/kaz_19jan2010/ | FAOSTAT^1^ |
| Kyrgyzstan | MED-Rice (2003). Guidance Document for Environmental Risk Assessments of Active Substances used on Rice in the EU for Annex I Inclusion. Document prepared by Working Group on MED-Rice, EU Document Reference SANCO/1090/2000 – rev.1, Brussels, June 2003, 108 pp. | FAOSTAT^1^ |
| Laos | Derived from ORYZA^7^ FAO: Food and Agriculture Organization of the United Nations, for a world without hunger. FAO Rice Information, Volume 3, December 2002. Retrieved October 31, 2012, from http://www.fao.org/docrep/005/Y4347E/y4347e00.htm#Contents Rice Almanac^6^ Reviewed by Ben Samson. | Lao Statistics.  FAOSTAT^1^ |
| Malaysia | Derived from ORYZA^7^ FAO: Food and Agriculture Organization of the United Nations, for a world without hunger. FAO Rice Information, Volume 3, December 2002. Retrieved October 31, 2012, from http://www.fao.org/docrep/005/Y4347E/y4347e00.htm#Contents Rice Almanac^6^ | Department of Agriculture Malaysia. http://www.doa.gov.my/web/guest/data_perangkaan_tanaman FAOSTAT^1^ |
| Myanmar | Calendar Myanmar for GIS by Khin Thawda Win, IRRI Post Doc, LIFT Project Ministry of Agriculture, Forestry and Fisheries. 2010. Status of annual production of rice by prefecture (translated from Japanese to English). Retrieved November 26, 2012 from http://www.maff.go.jp/j/tokei/kouhyou/sakumotu/sak kyou_kome/pdf/suitou_120815.pdf Reviewed by David Johnson. Reviewed by Nyo Me Htwe. | AFSIS (ASEAN Food Security Information System) http://afsis.oae.go.th/ FAO (Food and Agriculture Organization of the United Nations). 2013.  FAOSTAT^1^ |
| Nepal | FAO: Food and Agriculture Organization of the United Nations, for a world without hunger. FAO Rice Information, Volume 3, December 2002. Retrieved October 31, 2012, from http://www.fao.org/docrep/005/Y4347E/y4347e00.htm#Contents Gauchan, Devendra. Nepal rice calendar (unpublished). Gumma, M.K., Nelson, A., Thenkabail, P., Singh, A. Mapping rice areas of South Asia using MODIS multitemporal data. J. Appl. Remote Sens. 5(1), 053547. doi:10.1117/1.3619838 (2011). MVR Murty. Expert Opinion. | 2005 - FAORAP, RDES  2007 & 2009 - Government of Nepal Ministry of Agriculture and Cooperatives (http://www.moac.gov.np) FAOSTAT^1^ |
| North Korea | Boschetti, Mirco. Eurasia Rice Mapping Slide (unpublished). FAO: Food and Agriculture Organization of the United Nations, for a world without hunger. FAO Rice Information, Volume 3, December 2002. Retrieved October 31, 2012, from http://www.fao.org/docrep/005/Y4347E/y4347e00.htm#Contents MED-Rice (2003). Guidance Document for Environmental Risk Assessments of Active Substances used on Rice in the EU for Annex I Inclusion. Document prepared by Working Group on MED-Rice, EU Document Reference SANCO/1090/2000 – rev.1, Brussels, June 2003, 108 pp. | FAOSTAT^1^ |
| Pakistan | FAO: Food and Agriculture Organization of the United Nations, for a world without hunger. FAO Rice Information, Volume 3, December 2002. Retrieved October 31, 2012, from http://www.fao.org/docrep/005/Y4347E/y4347e00.htm#Contents  Gumma, M.K., Nelson, A., Thenkabail, P., Singh, A. Mapping rice areas of South Asia using MODIS multitemporal data. J. Appl. Remote Sens. 5(1), 053547. doi:10.1117/1.3619838 (2011). World Rice Statistics^5^ MVR Murty. Expert Opinion. | World Rice Statistics^5^  FAOSTAT^1^ |
| Philippines | Alosnos, E.D. and E.J.P. Quilang, comps. 2009-2010. GMA-RICE Program. Department of Agriculture IRRI. 1994-1999. SSD Farm Household Survey Database (RTDP). Malasa R., comps. 2010. Planting calendar of top rice-producing provinces (2004-2008). PhilRice Socio-economic Division Philippine Food Security Information System (PhilFSIS) crops. 2014 Philippine Statistics Authority (PSA). http://philfsis.psa.gov.ph/index.php/id/24 | PhilRice-BAS CountrySTAT Philippines http://countrystat.bas.gov.ph/ FAOSTAT^1^ |
| Saudi Arabia | World Rice Statistics^5^ | FAOSTAT^1^ |
| South Korea | Boschetti, Mirco. Eurasia Rice Mapping Slide (unpublished). MED-Rice (2003). Guidance Document for Environmental Risk Assessments of Active Substances used on Rice in the EU for Annex I Inclusion. Document prepared by Working Group on MED-Rice, EU Document Reference SANCO/1090/2000 – rev.1, Brussels, June 2003, 108 pp. | FAOSTAT^1^ |
| Sri Lanka | Gumma, M.K., Nelson, A., Thenkabail, P., Singh, A. Mapping rice areas of South Asia using MODIS multitemporal data. J. Appl. Remote Sens. 5(1), 053547. doi:10.1117/1.3619838 (2011). World Rice Statistics^5^ MVR Murty. Expert Opinion. | Agriculture and Environmental Statistics Division Department of Census and Statistics, Colombo, Sri Lanka FAOSTAT^1^ |
| Taiwan | World Rice Statistics^5^  Rice-Agriculture and Food Agency, Council of Agriculture, Executive Yuan. (n.d.). Introduction to Taiwan Rice. Retrieved October 24, 2012, from http://www.afa.gov.tw/content_en.aspx?hcatid=497&lcatid=494&pcatid=1&ycatid=1&scat=t | http://www.usda.gov/wps/portal/usda/usdahome FAOSTAT^1^ |
| Tajikistan | Boschetti, Mirco. Eurasia Rice Map (unpublished). | FAOSTAT^1^ |
| Thailand | Derived from ORYZA^7^ IRRI. 1994-1999. SSD Farm Household Survey Database (RTDP). Sawano, S., Hasegawa, T., Goto, S., Konghakote, P., Polthanee, A., Ishigooka, Y., et al. (2008). Modeling the dependence of the crop calendar for rain-fed rice on precipitation in Northeast Thailand. Paddy and Water Environment, 6(1), 83-89. Retrieved March 8, 2011, from http://link.springer.com/article/10.1007%2Fs10333-007-0102-x?LI=true Reviewed by David Johnson. | Agricultural Statistics of Thailand FAOSTAT^1^ |
| Turkey | Rice Almanac^6^  Reviewed by Neemi Beser.  Mirco Boschetti. Expert opinion. | FAOSTAT^1^ |
| Turkmenistan | Boschetti, Mirco. Eurasia Rice Map (unpublished). | FAOSTAT^1^ |
| Uzbekistan | Boschetti, Mirco. Eurasia Rice Mapping Slide (unpublished). | FAOSTAT^1^ |
| Vietnam | Derived from ORYZA^7^ Tung, D.T., comps. (n.d.). Rice in Vietnam (translated from Vietnamese to English). Retrieved November 16, 2012 from [www.khuyennongvn.gov.vn](http://www.khuyennongvn.gov.vn)  and <http://www.cuctrongtrot.gov.vn/>  Hijmans, Robert. Crop calendar data (unpublished). Htwe, N.M., trans. (n.d.). Planting and harvesting time in Myanmar (translated from Myanmar to English). Ministry of Agriculture and Irrigation. Retrieved from http://www.moai.gov.mm/ Tuong, TP (unpublished). | General Statistics Office of Vietnam http://www.gso.gov.vn/default_en.aspx?tabid=491 FAOSTAT^1^ |
| AFRICA |  |  |
| Algeria | Robert Hijmans. Crop calendar data (unpublished). | FAOSTAT^1^ |
| Angola | Global Information and Early Warning System (GIEWS) & FAO: Food and Agriculture Organization of the United Nations, for a world without hunger. 2012. GIEWS Country Briefs - homepage. <http://www.fao.org/giews/countrybrief/index.jsp>. Accessed on November 2015 | CountrySTAT^2^ FAOSTAT^1^ |
| Benin | FAO^3^ Cyriaque Akakpo. Expert opinion. AfricaRice and NARS partners. 2015 Review. | CountrySTAT^2^ FAOSTAT^1^ |
| Burkina Faso | FAO^3^ AfricaRice and NARS partners. 2015 Review. Zacharie Segda. Expert opinion. Louis Yameogo. Expert opinion. | CountrySTAT^2^ FAOSTAT^1^ |
| Burundi | FAO^3^ Joseph Bigirimana. Expert opinion.  AfricaRice and NARS partners. 2015 Review. | CountrySTAT^2^ FAOSTAT^1^ |
| Cameroon | FAO^3^ Ibrahim Bassoro. Expert opinion.  AfricaRice and NARS partners. 2015 Review. | FAOSTAT^1^  CountrySTAT^2^ |
| Central African Republic | FAO^3^ | FAOSTAT^1^ |
| Chad | FAO, 2002. FAO Rice Information, Volume 3, December 2002. Food and Agriculture Organization of the United Nations. Rome, Italy. Moundibaye Dastre Allarangaye. Expert opinion.  AfricaRice and NARS partners. 2015 Review. | FAOSTAT^1^ |
| Comoros | Robert Hijmans. Crop calendar data (unpublished). | FAOSTAT^1^ |
| Côte d'Ivoire | FAO^3^ Sander Zwart. Expert opinion. Henri Gbakatchetche. Expert opinion.  AfricaRice and NARS partners. 2015 Review. | FAOSTAT^1^  Rice Almanac 2011 datasets (Country_ricearea.mdb file) |
| Democratic Republic of the Congo | FAO^3^ Fanny Lunze. Expert opinion. AfricaRice and NARS partners. 2015 Review. | FAOSTAT^1^ |
| Egypt | FAO^3^ AfricaRice and NARS partners. 2015 Review. | FAOSTAT^1^ |
| Ethiopia | World Rice Statistics^5^ FAO^3^ Belay Abera Bayuh . Expert opinion. AfricaRice and NARS partners. 2015 Review. | FAOSTAT^1^ |
| Gabon | World Rice Statistics^5^ Yonnelle Moukoumbi. Expert opinion.  AfricaRice and NARS partners. 2015 Review. | FAOSTAT^1^ |
| Gambia | Carney, Judith A. Women's land right in Gambian irrigated rice schemes: Constraints and opportunities, Department of Geography, UCLA, April 2, 1998. Retrieved June 2014. http://link.springer.com/content/pdf/10.1023%2FA%3A1007580801416.pdf FAO^3^ Sander Zwart. Expert opinion. Famara Jaiteh. Expert opinion.AfricaRice and NARS partners. 2015 Review. | FAOSTAT^1^ Rice Almanac 2011 dataset using Robert Hijman's estimates and FAO and USDA rice area. |
| Ghana | FAO^3^ Raphael Kwame Bam. Expert opinion. Wilson Dogbe. Expert opinion. AfricaRice and NARS partners. 2015 Review. | CountrySTAT^2^ FAOSTAT^1^ |
| Guinea | FAO^3^ Kéita Sékou. Expert opinion.  AfricaRice and NARS partners. 2015 Review. | FAOSTAT^1^ |
| Guinea-Bissau | FAO, 2002. FAO Rice Information, Volume 3, December 2002. Food and Agriculture Organization of the United Nations. Rome, Italy. http://www.fao.org/docrep/005/Y4347E/y4347e00.htm#Contents | FAOSTAT^1^ Rice Almanac 2011 dataset using Robert Hijman's estimates and FAO and USDA rice. Updated by Andy Nelson, Stephan Haefele, and Sander Zwart. |
| Kenya | Robert Hijmans. Crop calendar data (unpublished). World Rice Statistics^5^ Rosemary Murori-Mutegi, Expert opinion.  AfricaRice and NARS partners. 2015 Review. | FAOSTAT^1^ |
| Lebanon | World Rice Statistics^5^ | FAOSTAT^1^ |
| Liberia | Sander Zwart. Expert opinion.  Buri Mohammed Moro. Expert opinion. FAO^3^ | FAOSTAT^1^ Rice Almanac 2011 dataset using Robert Hijman's estimates and FAO and USDA rice. Updated by Andy Nelson, Stephan Haefele, and Sander Zwart. |
| Madagascar | FAO^3^ Raymond Rabeson. Expert opinion.  AfricaRice and NARS partners. 2015 Review. | FAOSTAT^1^ |
| Malawi | FAO^3^ | FAOSTAT^1^ Rice Almanac 2011 dataset using Robert Hijman's estimates and FAO and USDA rice. Updated by Andy Nelson, Stephan Haefele, and Sander Zwart. |
| Mali | FAO^3^ Sander Zwart. Expert opinion. Nianankoro Kamissoko . Expert opinion. AfricaRice and NARS partners. 2015 Review. | CountrySTAT^2^ FAOSTAT^1^ |
| Mauritania | FAO^3^ Sander Zwart. Expert opinion. Habibou Gueye. Expert opinion. AfricaRice and NARS partners. 2015 Review. | FAOSTAT^1^ |
| Morocco | Boschetti, Mirco. Eurasia Rice Mapping Slide (unpublished). Sander Zwart. Expert opinion. | FAOSTAT^1^ |
| Mozambique | FAO^3^ Alexis Ndayiragije. Expert opinion.  AfricaRice and NARS partners. 2015 Review. | CountrySTAT^2^ FAOSTAT^1^ |
| Niger | FAO^3^ AfricaRice and NARS partners. 2015 Review. Illiassou Mossi Maïga, Adamou Bassou. Expert opinion. | CountrySTAT^2^ FAOSTAT^1^ |
| Nigeria | USDA, Crop calendar of Nigeria. URL: http://peaceworkspartners.org/vault/Nigeria/Research/Nigeria%20Statistics/crop_calendar_nigeria.pdf f. Accessed on October 31, 2012 Sander Zwart. Expert opinion. Oladele Samuel Bakare. Expert opinion. AfricaRice and NARS partners. 2015 Review. | CountrySTAT^2^ FAOSTAT^1^ |
| Republic of Congo | USDA. Crop calendar of Nigeria. URL:http://www.fas.usda.gov/pecad2/highlights/2002/03/nigeria/pictures/crop_calendar_nigeria.pdf. Accessed on October 31, 2012 AfricaRice and NARS partners. 2015 Review. | FAOSTAT^1^ |
| Reunion | FAO^3^ | FAOSTAT^1^ |
| Rwanda | Robert Hijmans. Crop calendar data (unpublished). Sander Zwart. Expert opinion. Alexis Ndayiragije. Expert opinion. Elie Rene Gasore, Alain Kalisa. Expert opinion. | CountrySTAT^2^ FAOSTAT^1^ |
| Senegal | FAO^3^ Sander Zwart. Expert opinion. Madiama Cisse. Expert opinion. AfricaRice and NARS partners. 2015 Review. | FAOSTAT^1^ Rice Almanac 2011 dataset using Robert Hijman's estimates and FAO and USDA rice. Updated by Andy Nelson, Stephan Haefele, and Sander Zwart. |
| Sierra Leone | World Rice Statistics^5^ Sander Zwart. Expert opinion. Idriss Baggie. Expert opinion. NARSinput data; Global Yield Gap Database (Dr. Kazuki Saito) | FAOSTAT^1^ Rice Almanac 2011 dataset using Robert Hijman's estimates and FAO and USDA rice. Updated by Andy Nelson, Stephan Haefele and Sander Zwart. |
| Somalia | FAO^3^ | FAOSTAT^1^ |
| South Africa | Robert Hijmans. Crop calendar data (unpublished). | FAOSTAT^1^ |
| Sudan | FAO^3^ | FAOSTAT^1^ |
| Swaziland | Robert Hijmans. Crop calendar data (unpublished). | FAOSTAT^1^ |
| Tanzania | FAO^3^ Geophrey  Kajiru . Expert opinion. AfricaRice and NARS partners. 2015 Review. | FAOSTAT^1^ Rice Almanac 2011 dataset using Robert Hijman's estimates and FAO and USDA rice. Updated by Andy Nelson, Stephan Haefele, and Sander Zwart. |
| Togo | Robert Hijmans. Crop calendar data (unpublished). Sander Zwart. Expert opinion. Komlan Adigninou Ablede, Essowedeou Sekou Ani. Expert opinion. AfricaRice and NARS partners. 2015 Review. | CountrySTAT^2^ FAOSTAT^1^ |
| Uganda | FAO^3^ David Nanfumba. Expert opinion.  AfricaRice and NARS partners. 2015 Review. | FAOSTAT^1^ Rice Almanac 2011 dataset using Robert Hijman's estimates and FAO and USDA rice area. |
| Zambia | FAO^3^ NARSinput data; Global Yield Gap Database (Kazuki Saito) | FAOSTAT^1^ Rice Almanac 2011 dataset using Robert Hijman's estimates and FAO and USDA rice. Updated by Andy Nelson, Stephan Haefele, and Sander Zwart. |
| Zimbabwe | Robert Hijmans. Crop calendar data (unpublished). | FAOSTAT^1^ |
| LATIN AMERICA AND THE CARIBBEAN | |  |
| Belize | FAO^4^ | FAOSTAT^1^ Rice Almanac 2011 dataset using Robert Hijman's estimates and FAO and USDA rice area. |
| Bolivia | FAO, 2002. FAO Rice Information, Volume 3, December 2002. Food and Agriculture Organization of the United Nations. Rome, Italy. http://www.fao.org/docrep/005/Y4347E/y4347e00.htm#Contents FAO^4^ World Rice Statistics^5^ | FAOSTAT^1^ |
| Brazil | FAO, 2002. FAO Rice Information, Volume 3, December 2002. Food and Agriculture Organization of the United Nations. Rome, Italy. http://www.fao.org/docrep/005/Y4347E/y4347e00.htm#Contents Rice Almanac^6^ The Brazilian Institute of Geography and Statistics (IBGE). Brazil Rice. URL: http://www.usda.gov/oce/weather/pubs/Other/MWCACP/Graphs/Brazil/BrzRiceProd_0509.pdf. Accessed on November 2015. | FAOSTAT^1^  Rice Almanac 2011 datasets (Country_ricearea.mdb file) |
| Chile | Viviana Lorena Becerra Velasquez. Expert opinion. | FAOSTAT^1^ Rice Almanac 2011 datasets (Country_ricearea.mdb file) |
| Colombia | FAO^4^ | FAOSTAT^1^ Rice Almanac 2011 datasets (Country_ricearea.mdb file) |
| Costa Rica | World Rice Statistics^5^ | FAOSTAT^1^ |
| Cuba | FAO, 2002. FAO Rice Information, Volume 3, December 2002. Food and Agriculture Organization of the United Nations. Rome, Italy. http://www.fao.org/docrep/005/Y4347E/y4347e00.htm#Contents | FAOSTAT^1^ |
| Dominican Republic | FAO, 2002. FAO Rice Information, Volume 3, December 2002. Food and Agriculture Organization of the United Nations. Rome, Italy. http://www.fao.org/docrep/005/Y4347E/y4347e00.htm#Contents Robert Hijmans. Crop calendar data (unpublished). | FAOSTAT^1^ |
| Ecuador | FAO^4^ | FAOSTAT^1^ Rice Almanac 2011 datasets (Country_ricearea.mdb file) |
| El Salvador | FAO^4^ Robert Hijmans. Crop calendar data (unpublished). | FAOSTAT^1^ Rice Almanac 2011 dataset using Robert Hijman's estimates and FAO and USDA rice area. |
| French Guiana | Robert Hijmans. Crop calendar data (unpublished). | FAOSTAT^1^ Rice Almanac 2011 dataset using Robert Hijman's estimates and FAO and USDA rice area. |
| Guatemala | FAO^4^ Robert Hijmans. Crop calendar data (unpublished). | FAOSTAT^1^ Rice Almanac 2011 dataset using Robert Hijman's estimates and FAO and USDA rice area. |
| Guyana | FAO^4^ | Guyana Rice Supply Chain Risk Assessment Report. February 2011. Available at https://www.agriskmanagementforum.org/sites/agriskmanagementforum.org/files/Documents/GuyanaRiceProd10.pdf FAOSTAT^1^ |
| Haiti | World Rice Statistics^5^ | FAOSTAT^1^ Rice Almanac 2011 dataset using Robert Hijman's estimates and FAO and USDA rice area. |
| Honduras | FAO^4^ Robert Hijmans. Crop calendar data (unpublished). | FAOSTAT^1^ Rice Almanac 2011 dataset using Robert Hijman's estimates and FAO and USDA rice area. |
| Jamaica | Robert Hijmans. Crop calendar data (unpublished). | FAOSTAT^1^ Rice Almanac 2011 dataset using Robert Hijman's estimates and FAO and USDA rice area. |
| Mexico | Mexican Rice Council. | FAOSTAT^1^ |
| Nicaragua | FAO, 2002. FAO Rice Information, Volume 3, December 2002. Food and Agriculture Organization of the United Nations. Rome, Italy. http://www.fao.org/docrep/005/Y4347E/y4347e00.htm#Contents | FAOSTAT^1^ Rice Almanac 2011 dataset using Robert Hijman's estimates and FAO and USDA rice area. |
| Panama | FAO^4^ | FAOSTAT^1^ Rice Almanac 2011 dataset using Robert Hijman's estimates and FAO and USDA rice area. |
| Paraguay | FAO, 2002. FAO Rice Information, Volume 3, December 2002. Food and Agriculture Organization of the United Nations. Rome, Italy. http://www.fao.org/docrep/005/Y4347E/y4347e00.htm#Contents FAO^4^ Robert Hijmans. Crop calendar data (unpublished). | FAOSTAT^1^ Rice Almanac 2011 dataset using Robert Hijman's estimates and FAO and USDA rice area. |
| Peru | FAO^4^ | FAOSTAT^1^ Rice Almanac 2011 datasets (Country_ricearea.mdb file) |
| Saint Lucia | World Rice Statistics^5^ | FAOSTAT^1^ |
| Suriname | FAO^4^ | FAOSTAT^1^  Rice Almanac 2011 dataset using Robert Hijman's estimates and FAO and USDA rice area. |
| Trinidad and Tobago | FAO^4^ | FAOSTAT^1^ Rice Almanac 2011 dataset using Robert Hijman's estimates and FAO and USDA rice area. |
| Uruguay | Gonzalo Carracelas (INIA Uruguay). Expert opinion.  FAO^4^ | FAOSTAT ^1^ Rice Almanac 2011 datasets (Country_ricearea.mdb file) |
| Venezuela | FAO^4^ | FAOSTAT^1^ Rice Almanac 2011 datasets (Country_ricearea.mdb file) |
| EUROPE |  |  |
| Bulgaria | Boschetti, Mirco and Nelson, Andy. Mapping rice areas in the temperate zones of Eurasia:  IRRI (unpublished). Hijmans, Robert. Crop calendar data (unpublished). | EUROSTAT  http://ec.europa.eu/eurostat/statistics-explained/index.php/Agriculture_statistics_at_regional_level#Cereals FAOSTAT^1^ |
| France | FAO: Food and Agriculture Organization of the United Nations, for a world without hunger. FAO Rice Information, Volume 3, December 2002. Retrieved October 31, 2012, from http://www.fao.org/docrep/005/Y4347E/y4347e00.htm#Contents Hijmans, Robert. Crop calendar data (unpublished). MED-Rice (2003). Guidance Document for Environmental Risk Assessments of Active Substances used on Rice in the EU for Annex I Inclusion. Document prepared by Working Group on MED-Rice, EU Document Reference SANCO/1090/2000 – rev.1, Brussels, June 2003, 108 pp. Reviewed by Mirco Boschetti. | EUROSTAT  http://ec.europa.eu/eurostat/statistics-explained/index.php/Agriculture_statistics_at_regional_level#Cereals FAOSTAT^1^ |
| Greece | FAO: Food and Agriculture Organization of the United Nations, for a world without hunger. FAO Rice Information, Volume 3, December 2002. Retrieved October 31, 2012, from http://www.fao.org/docrep/005/Y4347E/y4347e00.htm#Contents MED-Rice (2003). Guidance Document for Environmental Risk Assessments of Active Substances used on Rice in the EU for Annex I Inclusion. Document prepared by Working Group on MED-Rice, EU Document Reference SANCO/1090/2000 – rev.1, Brussels, June 2003, 108 pp.  Dimitrios Katsandonis (DEMETER) personal communication Reviewed by Mirco Boschetti. | EUROSTAT  http://ec.europa.eu/eurostat/statistics-explained/index.php/Agriculture_statistics_at_regional_level#Cereals FAOSTAT^1^ |
| Hungary | Boschetti, Mirco and Nelson, Andy. Mapping rice areas in the temperate zones of Eurasia:  IRRI (unpublished). Hijmans, Robert. Crop calendar data (unpublished). | EUROSTAT  http://ec.europa.eu/eurostat/statistics-explained/index.php/Agriculture_statistics_at_regional_level#Cereals FAOSTAT^1^ |
| Italy | FAO: Food and Agriculture Organization of the United Nations, for a world without hunger. FAO Rice Information, Volume 3, December 2002. Retrieved October 31, 2012, from http://www.fao.org/docrep/005/Y4347E/y4347e00.htm#Contents MED-Rice (2003). Guidance Document for Environmental Risk Assessments of Active Substances used on Rice in the EU for Annex I Inclusion. Document prepared by Working Group on MED-Rice, EU Document Reference SANCO/1090/2000 – rev.1, Brussels, June 2003, 108 pp. Reviewed by Massimo Biloni. | EUROSTAT  http://ec.europa.eu/eurostat/statistics-explained/index.php/Agriculture_statistics_at_regional_level#Cereals FAOSTAT^1^ |
| Portugal | FAO: Food and Agriculture Organization of the United Nations, for a world without hunger. FAO Rice Information, Volume 3, December 2002. Retrieved October 31, 2012, from http://www.fao.org/docrep/005/Y4347E/y4347e00.htm#Contents MED-Rice (2003). Guidance Document for Environmental Risk Assessments of Active Substances used on Rice in the EU for Annex I Inclusion. Document prepared by Working Group on MED-Rice, EU Document Reference SANCO/1090/2000 – rev.1, Brussels, June 2003, 108 pp. Reviewed by Mirco Boschetti. | EUROSTAT  http://ec.europa.eu/eurostat/statistics-explained/index.php/Agriculture_statistics_at_regional_level#Cereals FAOSTAT^1^ |
| Romania | Hijmans, Robert. Crop calendar data (unpublished).  Reviewed by Mirco Boschetti. | EUROSTAT  http://ec.europa.eu/eurostat/statistics-explained/index.php/Agriculture_statistics_at_regional_level#Cereals FAOSTAT^1^ |
| Russia | Boschetti, Mirco and Nelson, Andy. Eurasia crop establishment Rice Mapping  Hijmans, Robert. Crop calendar data (unpublished).  Rice Almanac^6^ | FAOSTAT^1^ |
| Spain | FAO: Food and Agriculture Organization of the United Nations, for a world without hunger. FAO Rice Information, Volume 3, December 2002. Retrieved October 31, 2012, from http://www.fao.org/docrep/005/Y4347E/y4347e00.htm#Contents Hijmans, Robert. Crop calendar data (unpublished). MED-Rice (2003). Guidance Document for Environmental Risk Assessments of Active Substances used on Rice in the EU for Annex I Inclusion. Document prepared by Working Group on MED-Rice, EU Document Reference SANCO/1090/2000 – rev.1, Brussels, June 2003, 108 pp. Reviewed by Mirco Boschetti. | EUROSTAT  http://ec.europa.eu/eurostat/statistics-explained/index.php/Agriculture_statistics_at_regional_level#Cereals FAOSTAT^1^ |
| Ukraine | USDA - JOINT AGRICULTURAL WEATHER FACILITY (JAWF). (n.d.). Ukraine: Rice. Retrieved January 1, 2012 from http://www.usda.gov/oce/weather/pubs/Other/MWCACP/Graphs/ukraine/ukraine_rice.pdf Hijmans, Robert. Crop calendar data (unpublished). Boschetti, Mirco. Eurasia Rice Mapping Slide (unpublished). | FAOSTAT^1^ |
| REST OF THE WORLD | |  |
| Australia | World Rice Statistics^5^ Reviewed by Russell Ford. | FAOSTAT^1^ |
| Fiji | Hijmans, Robert. Crop calendar data (unpublished). | F FAOSTAT^1^ |
| Papua New Guinea | FAO^3^ | FAOSTAT^1^ |
| Solomon Islands | Hijmans, Robert. Crop calendar data (unpublished). | FAOSTAT^1^ |
| USA | FAO: Food and Agriculture Organization of the United Nations, for a world without hunger. FAO Rice Information, Volume 3, December 2002. Retrieved October 31, 2012, from http://www.fao.org/docrep/005/Y4347E/y4347e00.htm#Contents Kent MacKenzie. Expert Opinion. | FAOSTAT^1^ |

^1^FAO. *FAOSTAT*. http://faostat.fao.org (2015).

^2^FAO. *CountrySTAT*. www.fao.org/economic/ess/countrystat/en/ (2015).

^3^FAO. *Crop calendar: an information tool for seed security*.. [www.fao.org/agriculture/seed/cropcalendar/welcome.do](http://www.fao.org/agriculture/seed/cropcalendar/welcome.do) (2010).

^4^FAO. *Calendario de cultivos: America Latina y el Caribe*. (FAO, 2006).

^5^IRRI. *World Rice Statistics* (Rice Crop Calendar by country, Appendix Table 4) (IRRI, 2008).

^6^Maclean, J.L., Dawe, D.C., Hardy, B., Hettel, G.P. *Rice Almanac, 3rd edn*. (International Rice Research Institute, 2002).

^7^Bouman, B. ORYZA2000, Version 2.13 (2009).
